# Prevalence and Antibiotic Resistance of *Streptococcus agalactiae* in Women of Childbearing Age Presenting Urinary Tract Infections from Western Romania

**DOI:** 10.3390/life14111476

**Published:** 2024-11-13

**Authors:** Constantin Catalin Marc, Monica Susan, Sergiu Adrian Sprintar, Monica Licker, Daniela Adriana Oatis, Daniela Teodora Marti, Razvan Susan, Laura Corina Nicolescu, Alin Gabriel Mihu, Tudor Rares Olariu, Delia Muntean

**Affiliations:** 1 Department of General Medicine, Doctoral School, “Victor Babes” University of Medicine and Pharmacy Timisoara, 300041 Timisoara, Romania; marc.catalin@yahoo.com; 2 Department of Biology and Life Sciences, Faculty of Medicine, Vasile Goldis Western University of Arad, 310025 Arad, Romania; danielaoatis@gmail.com (D.A.O.); dana_m73@yahoo.com (D.T.M.); laura_dsp@yahoo.com (L.C.N.); 3 Centre for Preventive Medicine, Department of Internal Medicine, “Victor Babes” University of Medicine and Pharmacy, Eftimie Murgu Square, No. 2, 300041 Timisoara, Romania; susan.monica@umft.ro; 4 “Aurel Ardelean” Institute of Life Sciences, Vasile Goldis Western University of Arad, 86 Rebreanu, 310414 Arad, Romania; sprintar.sergiu@gmail.com; 5 Multidisciplinary Research Center of Antimicrobial Resistance, Microbiology Department, “Victor Babes” University of Medicine and Pharmacy, 300041 Timisoara, Romania; licker.monica@umft.ro (M.L.); muntean.delia@umft.ro (D.M.); 6 Microbiology Laboratory, “Pius Brinzeu” County Clinical Emergency Hospital, 300723 Timisoara, Romania; 7 Faculty of Medicine, “Victor Babes” University of Medicine and Pharmacy from Timisoara, Eftimie Murgu Square No. 2, 300041 Timisoara, Romania; razvansusan@umft.ro; 8 Bioclinica Medical Analysis Laboratory, Dreptatii Street, nr. 23, 310300 Arad, Romania; 9 Discipline of Parasitology, Department of Infectious Disease, “Victor Babes” University of Medicine and Pharmacy, 300041 Timisoara, Romania; 10 Center for Diagnosis and Study of Parasitic Diseases, “Victor Babes” University of Medicine and Pharmacy, 300041 Timisoara, Romania; 11 Clinical Laboratory, Municipal Clinical Emergency Teaching Hospital, 300041 Timisoara, Romania

**Keywords:** group B *Streptococcus*, *Streptococcus agalactiae*, antibiotic resistance, women of childbearing age

## Abstract

Urinary tract infections (UTIs) are a common bacterial infection in women of childbearing age. *Streptococcus agalactiae* (Group B *Streptococcus*—GBS), a rare causative pathogen of UTIs in this population, is particularly important due to the potential risk during pregnancy, when it can lead to life-threatening neonatal infections. The current study analyzed 17,273 urine samples collected from consecutive women aged 18–45 years from Arad County, Western Romania. A total of 2772 samples tested positive for UTIs. In 130 cases, GBS was identified as the causative agent. Univariate logistic regression analysis revealed that women aged 25–34 years were more likely to test positive for GBS than those aged 18–24 years (cOR = 1.91, 95% CI: 1.07–3.43, *p* = 0.03). Antibiotic sensitivity testing revealed that all GBS strains were fully sensitive to penicillin, ampicillin, and vancomycin. High resistance was observed for clindamycin (77.34%) and tetracycline (88.46%). While GBS was found to be a rare pathogen in UTIs, our results underscore the importance of monitoring GBS in women of childbearing age, especially due to its risks during pregnancy, and emphasize the need for appropriate antibiotic management.

## 1. Introduction

Urinary tract infections (UTIs), defined as the presence of microorganisms in the urinary system [1,2], are among the most common bacterial infections, being a serious public health problem [3]. Women of childbearing age (15–44 years) have an increased vulnerability to UTI compared to men [4].

Group B *Streptococcus* (GBS) infection causes significant morbidity and mortality worldwide every year. According to the current medical literature, the prevalence of invasive infections caused by GBS is twice as high in pregnant women compared to non-pregnant women. The incidence of systemic invasive disease with GBS in pregnant women is 0.38 per 1000 pregnancies, with a mortality rate of 0.2% [5]. The rate of invasive GBS infections in newborns is 0.49 per 1000 live births [6]. *Streptococcus agalactiae* is a beta-hemolytic, Gram-positive bacterium [7] that colonizes the gastrointestinal and genital tracts as part of the normal human microbiota [8].

GBS can cause a range of severe invasive infections in vulnerable people, such as newborns, pregnant or postpartum women, the elderly, and individuals with compromised immune systems [8,9]. Pneumonia, sepsis, and meningitis are the most common diseases caused by GBS in newborns [10]. In adults, GBS can lead to bacteremia, pneumonia, osteoarthritis, skin infections, soft tissue infections, and urinary tract infections, including pyelonephritis and prostatitis [11,12,13].

GBS is a rare causative agent of UTIs [14]. UTIs caused by GBS are common in elderly, and immunocompromised individuals, pregnant women, patients with diabetes, and in those with pre-existing urologic abnormalities [15,16,17]. Newborns can become infected with GBS through several routes, such as exposure to vaginal secretions during birth or via breast milk [18,19]. Depending on when the newborn is infected with GBS after birth, invasive GBS disease is classified as early-onset invasive neonatal GBS disease (EOD) and late-onset disease (LOD) [20].

The primary route of transmission for neonatal infections is maternal vertical transmission [21]. EOD occurs within the first seven days of life and frequently presents with pneumonia and bacteremia [6,22]. The incidence of EOD in neonates born to GBS-carrier women is 29-fold higher than in those born to uncolonized women [23]. The bacterium can enter the newborn’s respiratory tract through amniotic fluid or contact with maternal colonization during vaginal delivery, causing lung tissue inflammation or GBS pneumonia [24].

LOD is an invasive neonatal infection with GBS that occurs between days 7 and 90 of the newborn’s life, frequently presenting with bacteremia, urinary tract infection, and meningitis [25,26]. Bacteria from the blood can cross the blood–brain barrier and migrate into the cerebrospinal fluid, causing neonatal meningitis. Occasionally, *Streptococcus agalactiae* can destroy the alveolar mucosa of the newborn via beta-hemolysin and reach the blood, causing bacteremia and neonatal sepsis [24]. Newborns with LOD are exposed to GBS through horizontal transmission. They usually acquire the same GBS serotype as their mothers’ colonizing strains [27]. In rare cases, bacteria can reach the joints through the bloodstream, favoring the onset of septic arthritis [24].

Prevention of maternal colonization with GBS is particularly important for the health of the newborn, reducing the incidence of early-onset symptomatic forms by 1–3% [28]. If, initially, the Center for Disease Control and Prevention (CDC) recommended screening pregnant women between 35 and 37 weeks of gestation to test for GBS colonization, the American College of Obstetricians and Gynecologists (2019) recommends antepartum screening for GBS between 36 0/7 and 37 6/7 weeks of gestation, a change from the previous testing interval [29]. GBS screening in pregnant women is essential for determining rectovaginal colonization and establishing intrapartum antibiotic prophylaxis (IAP), which helps reduce the risk of developing invasive neonatal infections [30].

Laboratory methods for GBS screening have not undergone major changes since 2010 [31]. According to the recommendations of the CDC, isolation and identification of biological samples of GBS should be performed in selective enrichment broths, such as Todd Hewitt broth, followed by microbial cultivation on 5% sheep blood agar, presumptive identification by the Christie-Atkins-Munch-Peterson (CAMP) test and confirmatory serogroup identification [32]. These recommendations are based on the fact that direct seeding of the rectovaginal sample on any culture medium, without prior passage through a selective enrichment broth, increases the chance of false negative results. Although the CDC mentions the use of chromogenic media for the identification of GBS, they cannot identify the non-hemolytic strains that represent up to 4% of GBS strains [32]

According to the 2020 American Society for Microbiology guidelines, culture remains the primary method for detecting GBS. Biochemical testing and latex agglutination are accepted methods for GBS identification. In the case of laboratories equipped with a matrix-assisted laser desorption ionization-time ionization-time-of-flight mass spectrometry (MALDI-TOF) instrument, protein-based identification is the ideal method for GBS analysis [31].

Due to the absence of large-scale screening procedures and lack of knowledge of GBS strains in Western Romania, we performed a retrospective analysis of positive isolates with *Streptococcus agalactiae* from females of childbearing age who presented for routine check-up and were found during the screening process with a UTI, assessing each strain’s antibiotic resistance profile.

## 2. Materials and Methods

### 2.1. Study Design and Setting

A retrospective cross-sectional study was performed on consecutive females of childbearing age (aged between 18 and 45 years) who presented for routine testing at Arad County Emergency Clinical Hospital and Bioclinica Laboratories between 1 January 2016 and 31 December 2022.

The study was conducted in Arad County, Western Romania. In 2021 the population of Arad County was estimated at 410,143 inhabitants. Arad is the capital city of Arad County, located in Western Romania with a population of 145,078. The rural population in Arad County represents approximately 45% of the total population of the county. The female population in Arad County represents approximately 210,941 (51.4%) of the total number of inhabitants [33].

### 2.2. UTI Definition, Sample Collection Inclusion, and Exclusion Criteria

In our study, UTIs were defined by the identification of a significant number of pathogenic bacteria, ≥10^5^ colony-forming units per milliliter (CFU/mL) on different culture mediums, in urine cultures coupled with the presence of leukocytes in the urine sediment examination.

**Figure 1:**
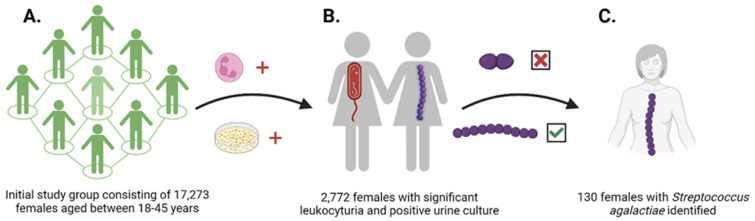
(**A**) A total of 17,273 females of childbearing age (18–45 years) were included in the study. (**B**) 2772 females presented with urinary tract infections, defined as the presence of microscopic leukocyturia and a >10^5^ CFU/mL culture on CHROMID^®^ CPS^®^ ELITE medium. (**C**) After identifying the *Streptococci* group (Group B *Streptococci*, excluding Group D *Streptococci*) and confirming *Streptococcus agalactiae* using Vitek 2, 130 females were selected.

A number of 17,273 clean midstream urine samples were collected from females of childbearing age after they were instructed to self-collect the midstream of urine using the clean-catch technique in a sterile container. Each study participant provided a single urine sample. From the initial sample pool, 2772 individuals were found to be present with a urinary tract infection. After identifying the pathogenic bacteria, 130 samples received from study participants were selected based on the growth of *Streptococcus agalactiae* on culture media (Figure 1). For the purpose of this study, healthy females were defined as individuals for whom UTIs were not diagnosed.

Exclusion criteria for the study included male patients; women outside the age range of 18–45 years; females with infections caused by microorganisms other than the study focus; individuals with inconclusive urine culture results (less than 10^5^ CFU/mL); participants with polymorphic flora; and females who were unwilling to participate.

### 2.3. Microscopic and Bacterial Examination

Urine microscopy examinations were conducted on all collected samples utilizing an automated urine microscopy analyzer, IRIS-iQ200 Series Analyzer (Beckman Coulter, Brea, CA, USA). Significant leukocyturia was considered >28 leukocytes/µL in accordance with the manufacturer’s instruction.

All collected samples were cultured with a calibrated loop designed to deliver 10 µL urine on CHROMID^®^ CPS^®^ ELITE (bioMérieux, Marcy-l’Étoile, France) in less than two hours after collection. The samples were incubated at 37 °C for 24 h.

**Figure 2:**
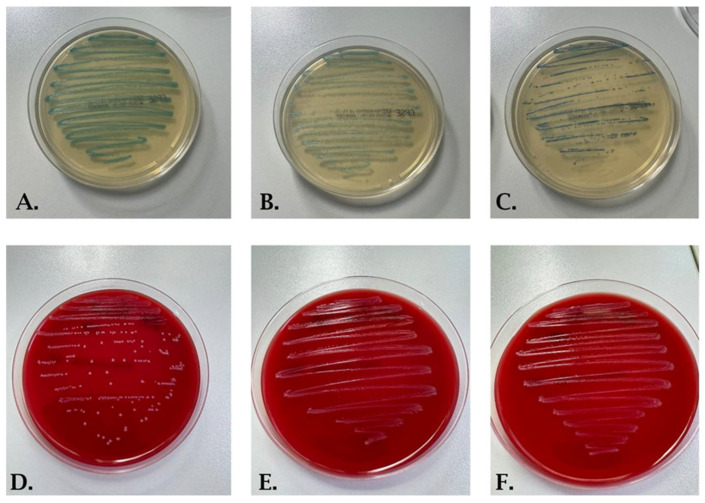
Cultures of *Streptococcus agalactiae* on CHROMID^®^ CPS^®^ ELITE (**A**–**C**) and blood agar medium (**D**–**F**).

All samples which presented on culture media monomorphic colonies developing over 10^5^ CFU/mL (Figure 2A–C) and significant leukocyturia were considered positive, and were proceeded to identify the microorganism. Suspect colonies of *Streptococcus* spp. were subcultured on a blood agar medium (Thermo Scientific™, Waltham, MA, USA) (Figure 2D–F). From the isolated colonies grown on the blood agar medium, we performed agglutination tests for identification of β-hemolytic streptococci (Pastorex Strep latex agglutination kit, Bio-Rad, Hercules, CA, USA) to differentiate group B streptococci from group D streptococci.

To further identify the species of Group B Streptococci, we utilized the Vitek^®^ 2 GP card (BioMérieux, Inc., Hazelwood, MO, USA) on the automated Vitek 2 Compact system (BioMérieux, Inc., Hazelwood, MO, USA). When the identification confidence level was 90% or higher (classified as very good or excellent), we proceeded to perform an antibiogram. For determining the bacterial sensitivity of the identified bacteria, we employed AST-ST03 cards (BioMérieux, Inc., Hazelwood, MO, USA), which included the following antibiotics: Penicillin, Ampicillin, Levofloxacin, Moxifloxacin, Erythromycin, Clindamycin, Linezolid, Teicoplanin, Vancomycin, Tetracycline, Tigecycline, Chloramphenicol, Rifampicin, and Trimethoprim + sulfamethoxazole. The results of the antibiogram were reported as follows: S for susceptible, I for intermediate, and R for resistant to the tested antibiotics. Both identification and antibiograms were interpreted by EUCAST guidelines and phenotypic interpretations in 2023 [34,35].

### 2.4. Data Collection and Statistical Analysis

Data were collected using Microsoft Excel, version 2011 (Microsoft Corp., Redmond, WA, USA). Statistical analyses were performed using Stata 16.1 (StataCorp, College Station, TX, USA). Data were presented as numbers, percentages, and mean ± standard deviation (SD).

Descriptive statistics were used to summarize the key characteristics of the study population. Mean and standard deviation were used for continuous variables, while percentage was used for categorical variables. Univariate logistic regression was used as the primary statistical model for this study. Crude odds ratios (cOR) with their corresponding 95% confidence intervals (95% CI) were presented for each statistical analysis. Statistical significance was set at *p* < 0.05.

### 2.5. Ethical Approval

This study was approved by the Ethics Committee of “Vasile Goldis” Western University of Arad, Romania (no. 30 from 5 June 2024) as well as the Ethics Committee of Arad County Emergency Clinical Hospital (no. 63 from 26 March 2024).

## 3. Results

Of the 17,273 (mean age 32 ± 6.76 years) urine samples obtained from females of childbearing age included in the study, 2772 (16.05%, 2772/17,273) were found with positive UTI test results, while 14,501 (83.95%, 14,501/17,273) were not diagnosed with UTI. Further analysis revealed that *Streptococcus agalactiae* was diagnosed in 130 (0.75%) of the 17,273 study participants and in 4.69% (130/2772) of those identified with UTI.

Univariate logistic regression analysis, comparing positive samples with *Streptococcus agalactiae* to samples obtained from healthy females revealed a statistically significant increase in the odds of testing positive for *Streptococcus agalactiae* in the 25–34 age group compared to the 18–24 age group (cOR = 1.91, 95% CI: 1.07–3.43, *p* = 0.03). When we compared females based on their area of residence (rural vs. urban), no significant relationship was observed (Table 1).

In this study, we evaluated the antibiotic susceptibility of *Streptococcus agalactiae* isolated from UTIs in females of childbearing age. All isolates were highly sensitive to penicillin (100%), ampicillin (100%), linezolid (100%), teicoplanin (100%), vancomycin (100%), tigecycline (100%), and trimethoprim-sulfamethoxazole (100%). Moxifloxacin also showed high sensitivity (94.62%), with only 5.38% resistance. However, significant resistance was observed for clindamycin (77.34%), tetracycline (88.46%), and erythromycin 35%). Levofloxacin had no fully sensitive isolates, with 90% showing intermediate sensitivity and 10% resistance. Chloramphenicol presented high sensitivity (97.44%) with minimal resistance (2.56%), while rifampicin showed intermediate results, with 53.85% sensitivity and 46.15% intermediate sensitivity (Table 2).

In the analysis comparing intermediate to resistant isolates for levofloxacin, urban residents were found to have 71% lower odds of developing resistant isolates compared to those living in rural areas, although this finding was not statistically significant (cOR = 0.29, 95% CI: 0.03–2.36, *p* = 0.25). When examining different age groups, individuals aged 25–34 years had a 97% lower chance of having resistant isolates, compared to those aged 18–24 years (cOR = 0.03, 95% CI: 0.001–1.05, *p* = 0.053), approaching statistical significance. Similarly, individuals aged 35–45 years showed a 67% reduction in odds of resistance, but this was also not statistically significant (cOR = 0.33, 95% CI: 0.14–8.18, *p* = 0.5).

For moxifloxacin, comparing sensible to resistant isolates, the data indicate that urban residents had a 23% reduction in the likelihood of resistance compared to rural residents, though this difference was not statistically significant (cOR = 0.77, 95% CI: 0.14–4.42, *p* = 0.77). In terms of age groups, individuals aged 25–34 years had an 81% lower chance of resistance compared to those aged 18–24 years (cOR = 0.19, 95% CI: 0.03–1.28, *p* = 0.09), and those aged 35–45 years had a 78% reduction in odds (cOR = 0.22, 95% CI: 0.02–2.69, *p* = 0.24). These findings were not statistically significant.

The analysis comparing sensible to resistant isolates for erythromycin showed that urban residents had 57% lower odds of resistance compared to rural residents, but this was not statistically significant (cOR = 0.43, 95% CI: 0.1–1.85, *p* = 0.26). When comparing age groups, those aged 35–45 years were over five times more likely to develop resistance compared to individuals aged 25–34 years, a significant finding (cOR = 5.2, 95% CI: 1.05–26.2, ***p* = 0.043**). Due to the small sample size in the 18–24 age group, statistical analysis for this group was not performed.

Regarding clindamycin, comparing sensible to resistant isolates, urban residents were found to be 40% less likely to have resistant isolates compared to rural residents, though this result was not statistically significant (cOR = 0.6, 95% CI: 0.22–1.62, *p* = 0.32). Age group analysis revealed that individuals aged 25–34 years were 75% less likely to have resistant isolates compared to those aged 18–24 years (cOR = 0.25, 95% CI: 0.3–2.04, *p* = 0.2), and those aged 35–45 years were 50% less likely (cOR = 0.5, 95% CI: 0.05–5.03, *p* = 0.56). None of these differences reached statistical significance.

For tetracycline, comparing sensible to resistant isolates, urban residents showed an 84% reduction in the likelihood of resistance compared to rural residents, which was approaching statistical significance (cOR = 0.16, 95% CI: 0.02–1.24, *p* = 0.08). However, age group analysis indicated a non-significant increase in resistance with age. Specifically, individuals aged 25–34 years had a 30% higher chance of resistance compared to those aged 18–24 years (cOR 1.3, 95% CI 0.25–6.69, *p* = 0.75), and those aged 35–45 years had a more than twofold increase in odds (cOR 2.27, 95% CI 0.28–18.27, *p* = 0.44).

In the case of rifampicin, comparing sensible to intermediate isolates, urban residents were found to have a 39% lower likelihood of resistance compared to rural residents, but this was not statistically significant (cOR 0.61, 95% CI 0.14–2.74, *p* = 0.52). Age group analysis showed that individuals aged 35–45 years had a 26% lower chance of resistance compared to those aged 25–34 years (cOR 0.74, 95% CI 0.15–3.69, *p* = 0.71), though this finding was also not statistically significant. The 18–24 age group was excluded from this analysis due to an insufficient sample size (Table 3).

## 4. Discussion

As with other studies [7,8,9,10], our research found a low prevalence of GBS as a causative agent for UTIs in females of childbearing age from Western Romania.

Although GBS colonization is asymptomatic and harmless in healthy women, it has been associated with severe infections in pregnant women [36,37,38,39,40]. Immunological, endocrinological, and metabolic changes during pregnancy can lead to significant alterations in the vaginal microbiome [41]. Typically, GBS is a harmless commensal of the vaginal microbiota and does not cause invasive diseases [42]. Vaginal colonization by GBS is inversely proportional to *Lactobacillus* populations in the vagina, which favors the creation of a dysbiotic environment with increased vaginal pH [42,43]. Such an environment is thought to favor the excessive multiplication of urogenital pathogens, such as GBS. As a transient vaginal pathobiont, GBS can shift from an asymptomatic carrier state in acidic conditions to an invasive state resistant to host immunity in neutral conditions. Fluctuations in pH from acidic to neutral upregulate the expression of several virulence genes, facilitating the transition of GBS to a virulent state [44]. One of the most important virulence factors is beta-hemolysin/cytolysin [45], which plays a key role in the dissemination of GBS in uterine, placental, and fetal tissues during pregnancy [46]. GBS frequently colonizes the gastrointestinal and genitourinary tracts and can also be found in the oropharynx [47,48,49].

Penicillin is a beta-lactam antibiotic and is currently the first-line treatment for the prevention of GBS-EOD and for treating GBS infections [32,50,51,52,53]. However, GBS strains with reduced sensitivity to penicillin have been reported in Hong Kong [54], Japan [55,56], the United States [57], and South Korea [58]. Beta-lactam antibiotics exert their antibacterial effect by inhibiting the action of enzymes involved in cell wall synthesis [59].

Ampicillin belongs to the aminopenicillin category, one of the five classes of penicillins [60]. In our study, all clinical isolates analyzed were found to be sensitive to penicillin and ampicillin. In a study conducted in Ethiopia, ampicillin resistance was noted in 10.4% of cases [61], whereas in Zimbabwe, it was 58.1% [62]. For patients allergic to penicillin and for whom second-line antibiotics are ineffective, vancomycin is recommended [59].

Macrolides (such as erythromycin) and lincosamides (clindamycin) are recommended as second-line antibiotics for patients with beta-lactam allergy [29,32]. The emergence of resistance to penicillin, erythromycin, and clindamycin in *Streptococcus agalactiae* isolates has raised significant concerns regarding the use of these antibiotics [63,64], with GBS antibiotic resistance becoming a global issue [7]. Resistance to macrolides and clindamycin among invasive GBS isolates has increased over the past two decades, from less than 5% to 20–30% [50,53]. The rise in resistance to these antibiotics has led to limitations in their use [29,32]. In our study, we observed that 35% of isolates showed resistance to erythromycin, a percentage similar to that reported in Portugal [65]. This result is close to the rates observed in France (34.7%) ([66], Italy (32.20%) [67], and Switzerland (30%) [68]. In a recent study conducted in Romania, Petca and colleagues (2024) observed an erythromycin resistance rate of 25% among pregnant women [69]. Similar results have been reported in Sri Lanka, with an erythromycin resistance rate of 24.4% [70], and in Ethiopia at 23.88% [61]. However, erythromycin resistance in GBS strains was significantly higher in China, where rates of 70% [52], 74.1% [71], and 78.6% [72] have been reported. High resistance rates have also been reported in countries such as Vietnam (76.23%) [73], Iran (73.7%) [74], Taiwan (68.1 [75], Nigeria (64.7%) [76], Senegal (53.5%) [77], and the United States (44.8%) [20].

Clindamycin is an antibiotic from the lincosamide class [78] and is the main antimicrobial agent indicated for patients with penicillin allergy [79]. There has been a constant increase in clindamycin resistance worldwide among GBS strains [21,59]. In our study, the resistance rate to clindamycin was 77.34%. A higher percentage of resistance to clindamycin was reported in Ethiopia (88%) [61]. In other countries, clindamycin resistance was lower compared to this study. In China, clindamycin resistance ranged between 64.2 and 72.1%: 64.2% [71], 64.3% [72], and 72.1% [52]. High levels of clindamycin resistance were observed in Taiwan (65.9%) [75], Vietnam (58.21%) [73], Zimbabwe (55.8%) [62], and Iran (52.6%) [74]. In other countries, the percentage of clindamycin resistance was lower, namely 43.75% in Italy [67], 34% in Portugal [65], 30.4% in Kenya [80], 28% in Switzerland [68], 26.8% in Ethiopia [81], 25.6% in Senegal [77], 23.7% in Romania [69], 22.2% in Sri Lanka [70], and 20.8% in the USA [20]. In the USA, from 2006 to 2015, there was a significant increase in the resistance rate to erythromycin from 34.7% to 49.1%, while clindamycin resistance increased from 14.7% to 26.0% [20]. In the UK, clindamycin is no longer recommended against GBS due to the high resistance to this antibiotic [51].

Levofloxacin is a second-generation fluoroquinolone antibiotic that shows improved activity against Gram-positive bacteria [59]. Moxifloxacin belongs to the third generation of fluoroquinolones and has superior antibacterial activity [82]. In our study, we observed 10% resistance to levofloxacin and 5.38% resistance to moxifloxacin. In contrast, a higher resistance rate to levofloxacin and moxifloxacin (28.6%) was reported in China in GBS isolates from pregnant women with premature rupture of membranes [52]. Compared to this study, higher resistance rates to levofloxacin (18.6%) and moxifloxacin (16.3%) were reported in pregnant women in Senegal [77]. Van Du et al. (2021) reported a levofloxacin resistance rate of 28.46% in pregnant women in Vietnam [73], while Dashtizadeh et al. (2020) reported a 21.1% resistance rate to levofloxacin in Iran [74].

There have been few studies regarding resistance to linezolid in clinical isolates of *S. agalactiae*. In this study, no resistance to linezolid was noted, similar to the survey conducted by Ngom et al. (2023) in Senegal [77]. However, the presence of linezolid-resistant *S. agalactiae* strains among clinical isolates has been observed in China [83] and, more recently, in Romania [69]. In this study, resistance to teicoplanin was 0%, with similar results obtained in Senegal [77].

The glycopeptide antibiotic, vancomycin, has been considered for several decades the last resort for treating severe infections caused by Gram-positive bacteria [84]. It is used in the treatment of GBS infections as well as in IAP [29,32,51]. Vancomycin is indicated for GBS-colonized mothers who are at high risk of anaphylactic shock from penicillin and when the bacterial isolate is resistant to second-line antibiotics [32,79]. No resistance to vancomycin was observed in this study. There are few studies regarding vancomycin resistance in GBS. In the USA, two cases of invasive GBS infection resistant to vancomycin have been reported [85]. A meta-analysis conducted by Gizachew et al. (2019) highlighted vancomycin resistance in GBS strains isolated from pregnant women in Africa [86]. The percentage of vancomycin resistance varied from 16.41% in Ethiopia [61] to 17% in Ethiopia [81], 24.1% in Kenya [80], and 70.6% in Nigeria [76].

Tetracyclines are bacteriostatic antibiotics (Grossman, 2016) that inhibit protein synthesis by preventing the attachment of aminoacyl-tRNA to the ribosomal acceptor (A) site [87]. The excessive use of tetracycline has led to the emergence of resistance to this antibiotic [59]. Resistance to tetracycline in GBS is often high, frequently exceeding 80%, and multiple resistance determinants can be identified in GBS strains [88,89,90]. In this study, the resistance to tetracycline in clinical isolates was 88.46%, a value similar to that reported in Ethiopia (88%) [61], Vietnam (89.66%) [73], and China (83.9%) [72] and 81.4% [52]. In contrast, among pregnant women in Senegal, a higher resistance rate of 100% was observed [77], and in Zimbabwe, the rate was 97.7% [62]. The susceptibility of *Streptococcus agalactiae* isolates to tetracycline was 11.54% in this study, a result close to that reported by Kumalo et al. (2023) in Ethiopia, where a susceptibility of 8.9% was recorded [61].

Tigecycline is a semi-synthetic antibacterial agent from the tetracycline class, developed for the treatment of polymicrobial infections caused by multidrug-resistant Gram-positive and Gram-negative pathogens [91]. Tigecycline could be considered an alternative to penicillin for the treatment of infections caused by erythromycin-resistant *S. agalactiae* [92,93]. In this study, the resistance to tigecycline was 0%, similar to the study conducted by Ngom et al. (2023) in Senegal [77].

We found that the resistance to chloramphenicol was 2.56%. A similar value of 2.3% was observed in Senegal [77]. In other countries, higher values of resistance to chloramphenicol have been reported. In China, it was 12.9% [94], in Zimbabwe 34.9% [62], in Iran 42.1% [74], and in Vietnam 52.38% [73]. In this study, the susceptibility of GBS isolates to chloramphenicol was 97.44%, which is similar to the 92.5% reported by Kumalo et al. (2023) in Ethiopia (Kumalo et al., 2023) [61].

In this study, no resistance to trimethoprim + sulfamethoxazole was observed. Previously, resistance to trimethoprim + sulfamethoxazole was reported in Brazil in GBS isolates from pregnant women (17.95%) [95].

Our study acknowledges certain limitations: (I) the overrepresentation of women aged 25–34 years and those from urban areas; (II) the lack of information about the pregnancy status of the participants; (III) the absence of selective enrichment broths, such as Todd Hewitt broth; (IV) the lack of confirmation of Vitek identification with MALDI-TOF; (V) incomplete analysis of some antibiotics across all participants; (VI) we not using questionnaires to distinguish between symptomatic infection and asymptomatic bacteriuria; and (VII) we not controlling for the increase in familywise error rates throughout the statistical analysis. Our findings should be considered preliminary, and we encourage further replication.

## 5. Conclusions

While GBS remains a rare pathogen in the case of UTIs, our data bring new information on the prevalence and antibiotic resistance profile of GBS isolated from females of childbearing age with UTIs from Western Romania. We reported that GBS isolates were fully sensitive to penicillin, ampicillin, and vancomycin. However, high levels of resistance were observed for clindamycin and tetracycline.

Considering the impact GBS can have on the newborn, policymakers should consider adopting large-scale screening procedures. Our results also highlight the need for targeted educational interventions to increase awareness regarding the importance of GBS screening in women of childbearing age. The adoption of such measures can contribute to the improvement of impro maternal and neonatal health outcomes.

## Figures and Tables

**Table 1:** Age distribution and area of residence of healthy females and females infected with *Streptococcus agalactiae* from Western Romania.

Age Group (%)	Females Infected with *Streptococcus agalactiae* (n = 130) (%)	Healthy Females (n = 14,501) (%)	cOR	95% CI	*p*-Value
18–24 years (100)	13 (0.63)	2052 (99.37)		Ref.	
25–34 years (100)	90 (2.20)	7431 (98.90)	1.91	1.07–3.43	0.03
34–45 years (100)	27 (0.54)	5018 (99.46)	0.85	0.44–1.65	0.63
**Area of residence (%)**					
Rural (100)	37 (0.91)	4014 (99.09)		Ref.	
Urban (100)	93 (0.88)	10,487 (99.12)	0.96	0.66–1.41	0.84

Ref., reference; n, total number.

**Table 2:** Antibiotic susceptibility of *Streptococcus agalactiae* isolated from urinary tract infections in females of childbearing age from Western Romania.

Antibiotic (Total, 100%)	Sensible (%)	Intermediate (%)	Resistant (%)
Penicillin (n = 130)	130 (100%)	0 (0%)	0 (0%)
Ampicillin (n = 88)	88 (100%)	0 (0%)	0 (0%)
Levofloxacin (n = 40)	0 (0%)	36 (90%)	4 (10%)
Moxifloxacin (n = 130)	123 (94.62%)	0 (0%)	7 (5.38%)
Erythromycin (n = 40)	26 (65%)	0 (0%)	14 (35%)
Clindamycin (n = 128)	29 (22.66%)	0 (0%)	99 (77.34%)
Linezolid (n = 127)	127 (100%)	0 (0%)	0 (0%)
Teicoplanin (n = 69)	69 (100%)	0 (0%)	0 (0%)
Vancomycin (n = 122)	122 (100%)	0 (0%)	0 (0%)
Tetracycline (n = 130)	15 (11.54%)	0 (0%)	115 (88.46%)
Tigecycline (n = 122)	122 (100%)	0 (0%)	0 (0%)
Chloramphenicol (n = 39)	38 (97.44%)	0 (0%)	1 (2.56%)
Rifampicin (n = 39)	21 (53.85%)	18 (46.15%)	0 (0%)
Trimethoprim + sulfamethoxazole (n = 130)	130 (100%)	0 (0%)	0 (0%)

**Table 3:** Univariate analysis assessing potential links between age groups and area of residence and various antibiotics to which GBS isolates from females of childbearing age with UTIs from Western Romania.

Antibiotic		cOR	95% CI	*p*-Value
Levofloxacin **	Age group			
	18–24 years	Ref.		
	25–34 years	0.03	0.001–1.05	0.053
	34–45 years	0.33	0.14–8.18	0.5
	Area of residence			
	Rural	Ref.		
	Urban	0.29	0.03–2.36	0.25
Moxifloxacin *	Age group			
	18–24 years	Ref.		
	25–34 years	0.19	0.03–1.28	0.09
	34–45 years	0.22	0.02–2.69	0.24
	Area of residence			
	Rural	Ref.		
	Urban	0.77	0.14–4.42	0.77
Erythromycin *	Age group			
	18–24 years	N/A.		
	25–34 years	Ref.		
	34–45 years	5.2	1.05–26.2	0.043
	Area of residence			
	Rural	Ref.		
	Urban	0.43	0.1–1.85	0.26
Clindamycin *	Age group			
	18–24 years	Ref.		
	25–34 years	0.25	0.3–2.04	0.2
	34–45 years	0.5	0.05–5.03	0.56
	Area of residence			
	Rural	Ref.		
	Urban	0.6	0.22–1.62	0.32
Tetracycline *	Age group			
	18–24 years	Ref.		
	25–34 years	1.3	0.25–6.69	0.75
	34–45 years	2.27	0.28–18.27	0.44
	Area of residence			
	Rural	Ref.		
	Urban	0.16	0.02–1.24	0.08
Rifampicin ***	Age group			
	18–24 years	N/A.		
	25–34 years	Ref.		
	34–45 years	0.74	0.15–3.69	0.71
	Area of residence			
	Rural	Ref.		
	Urban	0.61	0.14–2.74	0.52

N/A., not applicable. Ref., reference. *, sensible strains were compared to resistant ones. **, intermediate strains were compared to resistant ones. ***, sensible strains were compared to intermediate ones.

## Data Availability

Data are contained within the article.
